# Danger of Mistaking a Detained Testicle for Bubonocele

**Published:** 1846-05

**Authors:** 


*Danger of mistaking a detained Testicle for Bubonocele.*—If one of the patient's testicles should never have descended into the scrotum, but remains in the inguinal canal, and this testicle gets inflamed from any injury, it gives precisely the symptoms of strangulated hernia; I would defy any one to tell the difference between them. Now, if you proceed to operate in this case under the impression that it is hernia, you will perform an operation that will be very likely to be fatal to the patient; merely exposing the testicle in this situation is almost always fatal. I recollect I once went to perform an operation of this kind, and did perform it, without ever thinking whether the man had a scrotum or not. I found the symptoms arose from an inflamed testicle in the canal. The man died, and the case left an impression on my mind that I will not readily forget. Inflammation of the testicle here is infinitely more violent than it is in the scrotum, and when it is exposed the patient dies of peritoneal inflamation.—*Ibid.*

